# Effect of vitamin D supplementation on fatigue and mood among perimenopausal women

**DOI:** 10.6026/973206300221638

**Published:** 2026-03-31

**Authors:** Prama Ray, Arshi Srivastava, Parth Jani

**Affiliations:** 1 Department of Obstetrics and Gynaecology, Jagannath Gupta Institute of Medical Sciences, North Kolkata Campus, India; 2 Department of Biochemistry, Era Medical College, Lucknow, Uttar Pradesh, India; 3 Department of General Medicine, Government Medical College, Bhavnagar, Gujarat, India

**Keywords:** Perimenopause, vitamin D supplementation, fatigue, mood disorders, women's health

## Abstract

Perimenopausal women often experience fatigue and mood disturbances, which may be associated with vitamin D deficiency. Therefore, it is of interest to investigate the effect of vitamin D supplementation on fatigue and mood in perimenopausal women with vitamin D deficiency. A randomized, double-blind, placebo-controlled trial included 100 women who received either vitamin D3 or a placebo for 6 months. Results showed significant improvements in both fatigue and mood in the vitamin D group compared to placebo. Multivariate analysis confirmed vitamin D supplementation as a predictor of these improvements. Thus, vitamin D supplementation can effectively alleviate fatigue and improve mood in perimenopausal women with vitamin D deficiency.

## Background:

Perimenopause represents a critical transitional phase in a woman's life, typically occurring in the years preceding menopause and characterized by complex hormonal fluctuations, particularly in estrogen and progesterone levels [1]. This period is often accompanied by a wide range of physical, psychological and emotional symptoms that can substantially impair quality of life. Among these symptoms, fatigue and mood disturbances such as irritability, low mood, anxiety and depressive symptoms are among the most frequently reported and distressing complaints. These manifestations not only affect daily functioning and productivity but also contribute to long-term health consequences, including reduced social engagement, impaired occupational performance and increased healthcare utilization [2]. Fatigue during perimenopause is multifactorial in origin. Hormonal variability, sleep disturbances due to vasomotor symptoms, metabolic changes and psychosocial stressors all contribute to persistent tiredness and low energy levels. Similarly, mood changes during this stage are influenced by neuroendocrine alterations, heightened stress reactivity and changes in neurotransmitter systems [3]. Although these symptoms are often attributed solely to hormonal shifts, emerging evidence suggests that nutritional deficiencies may play a significant and potentially modifiable role in the pathophysiology of perimenopausal fatigue and mood disorders [4]. Vitamin D, a fat-soluble secosteroid hormone, has traditionally been recognized for its essential role in calcium homeostasis and bone metabolism. However, over the past two decades, research has increasingly highlighted its pleiotropic effects on multiple organ systems, including the immune, muscular and central nervous systems [5]. Vitamin D receptors and the enzyme responsible for its activation are widely distributed in brain regions involved in mood regulation, such as the prefrontal cortex, hippocampus and limbic system. These findings suggest that vitamin D may influence neurocognitive and emotional processes, making it a biologically plausible factor in mood and fatigue regulation [6]. Vitamin D deficiency is highly prevalent among perimenopausal women worldwide, particularly in developing countries and regions with limited sun exposure, cultural clothing practices, indoor lifestyles and dietary insufficiency [7]. Aging-related changes in skin synthesis, reduced outdoor activity and altered metabolism further exacerbate the risk of deficiency during midlife.

Low vitamin D levels have been associated with musculoskeletal pain, reduced muscle strength and generalized fatigue, all of which may compound the physical burden experienced during perimenopause. Moreover, deficiency has been linked to depressive symptoms, anxiety and cognitive impairment in various populations, suggesting a broader impact on mental well-being [8]. The relationship between vitamin D status and mood disorders has garnered increasing scientific interest. Proposed mechanisms include vitamin D's role in regulating inflammatory cytokines, modulating serotonin synthesis and influencing hypothalamic-pituitary-adrenal (HPA) axis activity. Chronic low-grade inflammation, which is common during the menopausal transition, has been implicated in the development of fatigue and depression [9]. Vitamin D's anti-inflammatory properties may therefore be particularly relevant in alleviating these symptoms. Additionally, vitamin D may improve sleep quality and neuromuscular function, indirectly contributing to reduced fatigue and improved mood [10]. Despite growing evidence linking vitamin D deficiency with fatigue and mood disturbances, findings from interventional studies remain inconsistent. While some randomized controlled trials have demonstrated improvements in depressive symptoms and fatigue following vitamin D supplementation, others have reported minimal or no benefit [11]. These discrepancies may be attributed to variations in study populations, baseline vitamin D status, dosage and duration of supplementation, outcome measures and confounding factors such as physical activity and comorbid conditions. Importantly, perimenopausal women constitute a unique population with distinct hormonal and psychosocial characteristics, yet they remain under represented in vitamin D intervention research [12]. Current management strategies for perimenopausal fatigue and mood changes often focus on hormone replacement therapy, antidepressants or lifestyle modifications. However, not all women are suitable candidates for hormonal or pharmacological treatments due to contraindications, side effects or personal preferences. Nutritional interventions, including vitamin D supplementation, offer a potentially safe, cost-effective and accessible adjunct or alternative approach [13]. Establishing clear evidence for the efficacy of vitamin D supplementation in improving fatigue and mood could have significant clinical and public health implications, particularly in resource-limited settings [14]. Given the high prevalence of vitamin D deficiency, the substantial burden of fatigue and mood disturbances during perimenopause and the biological plausibility linking vitamin D to neuropsychological health, it is essential to further explore this relationship through well-designed clinical studies [15]. Therefore, it is of interest to investigate the effect of vitamin D supplementation on fatigue and mood in perimenopausal women, as it may provide valuable insights into a simple and effective therapeutic intervention for managing these common symptoms.

## Methodology:

## Study design and setting:

This original research was designed as a prospective, randomized, double-blind, placebo-controlled interventional study conducted at a tertiary care hospital/community health center over a period of 6 months. The study aimed to evaluate the effect of vitamin D supplementation on fatigue and mood among perimenopausal women.

## Sample size:

A total sample size of 100 perimenopausal women was included in the study. The sample size was determined based on previous literature indicating a moderate effect size of vitamin D supplementation on fatigue and mood scores, with a confidence level of 95% and a power of 80%, allowing for a 10% attrition rate.

## Study population:

Women aged 40-55 years who were in the perimenopausal phase, defined by irregular menstrual cycles and/or climacteric symptoms were screened for eligibility.

## Inclusion criteria:

[1] Perimenopausal women aged 40-55 years

[2] Complaints of fatigue and/or mood disturbances for at least 3 months

[3] Serum 25-hydroxyvitamin D levels <30 ng/mL

[4] Willingness to participate and provide written informed consent

## Exclusion criteria:

[1] Postmenopausal women or women on hormone replacement therapy

[2] Current use of antidepressants, anxiolytics or vitamin D supplementation within the past 3 months

[3] Known psychiatric disorders, thyroid disease, chronic kidney or liver disease

[4] Malabsorption syndromes or metabolic bone disorders

[5] Pregnancy or lactation

## Randomization and blinding:

Eligible participants were randomly allocated into two groups (n = 50 each) using a computer-generated randomization sequence. Allocation concealment was ensured using sealed opaque envelopes. Both participants and investigators were blinded to group allocation.

[1] Intervention Group (Group A): Received oral vitamin D3 supplementation

[2] Control Group (Group B): Received a matching placebo

## Intervention:

Participants in the intervention group received oral vitamin D_3_ at a dose of 60,000 IU once weekly for 8 weeks, followed by 60,000 IU once monthly for the subsequent 4 months. The control group received an identical placebo on the same schedule. Compliance was assessed through pill counts and participant self-reporting.

## Data collection and outcome measures:

Baseline data including age, body mass index (BMI), menstrual history, sun exposure, dietary habits and physical activity were recorded. Serum 25-hydroxyvitamin D levels were measured at baseline and at the end of the study period. Fatigue was assessed using a validated fatigue assessment scale, while mood was evaluated using a standardized mood or depression rating scale. Assessments were conducted at baseline, at 8 weeks and at 6 months.

## Ethical considerations:

The study protocol was approved by the Institutional Ethics Committee. Written informed consent was obtained from all participants prior to enrollment. Confidentiality and anonymity of participants were maintained throughout the study.

## Statistical analysis:

Data were analyzed using statistical software. Continuous variables were expressed as mean ± standard deviation and categorical variables as frequencies and percentages. Within-group comparisons were performed using paired t-tests or Wilcoxon signed-rank tests, while between-group comparisons were analyzed using independent t-tests or Mann-Whitney U tests. A p-value <0.05 was considered statistically significant.

## Results:

A total of 100 perimenopausal women were enrolled and randomized equally into the vitamin D supplementation group (n = 50) and the placebo group (n = 50). All participants completed the study and their data were included in the final analysis. At baseline, both groups were comparable with respect to age, body mass index (BMI), serum 25-hydroxyvitamin D levels, fatigue scores and mood scores, with no statistically significant differences observed (p > 0.05), indicating successful randomization (Table 1 - see PDF). After 6 months, the vitamin D group showed a significant increase in mean serum 25(OH)D levels compared to baseline (p < 0.001), whereas no significant change was observed in the placebo group (Table 2 - see PDF). A significant reduction in fatigue scores was observed in the vitamin D group at both 8 weeks and 6 months compared to baseline (p < 0.001). In contrast, the placebo group showed no statistically significant improvement. Between-group comparison at 6 months demonstrated significantly lower fatigue scores in the vitamin D group (p < 0.001) (Table 3 - see PDF). Mood scores improved significantly in the vitamin D group over the study period, with a marked reduction in depressive symptoms at 6 months (p < 0.001). No significant change was observed in the placebo group. Between-group analysis revealed a statistically significant difference favoring vitamin D supplementation (Table 4 - see PDF). A multivariate linear regression analysis was performed using STATA to assess the independent effect of vitamin D supplementation on fatigue and mood scores at 6 months after adjusting for age, BMI, baseline vitamin D levels and sun exposure. The regression analysis demonstrated that vitamin D supplementation was an independent and significant predictor of improvement in both fatigue and mood scores at 6 months (p < 0.001), even after controlling for potential confounders (Table 5 - see PDF). Overall, the results indicate that vitamin D supplementation significantly improves serum vitamin D status, reduces fatigue and enhances mood among perimenopausal women compared to placebo (Table 2-5 - see PDF).

## Discussion:

The present study demonstrates that vitamin D supplementation leads to a significant improvement in both fatigue and mood symptoms among perimenopausal women when compared with placebo. After six months of intervention, women receiving vitamin D showed marked reductions in fatigue scores and depressive mood scores, even after adjusting for potential confounders such as age, body mass index and baseline vitamin D levels. These findings suggest that correction of vitamin D deficiency may play a meaningful role in alleviating common and often debilitating symptoms experienced during the perimenopausal transition. Our findings are consistent with several previous studies that have explored the relationship between vitamin D supplementation, fatigue and mood-related outcomes. Penckofer *et al.* (2017) [16] conducted a randomized controlled trial evaluating the effects of vitamin D supplementation on depressive symptoms in women with low baseline vitamin D levels. They reported a significant reduction in depression and anxiety scores following supplementation over a six-month period. This aligns closely with our results, as both studies demonstrate that improving vitamin D status is associated with better mood outcomes, particularly in women experiencing hormonal or metabolic vulnerability. Similarly, Fazelian *et al.* (2019) [17] examined the impact of vitamin D supplementation on psychological outcomes in women and found significant improvements in mood parameters after supplementation. Their study also suggested that vitamin D's anti-inflammatory properties may contribute to its beneficial psychological effects. These findings support our observation that vitamin D supplementation independently predicted improvement in mood scores, even after controlling for confounding variables. In another interventional study, Heidari *et al.* (2024) [18] assessed the effect of vitamin D supplementation on mood disturbances in vitamin D deficient women and reported a significant reduction in depressive symptoms following treatment. Although their population differed slightly from ours, the direction and magnitude of mood improvement were comparable. This suggests that the beneficial psychological effects of vitamin D may extend across different female life stages, including the perimenopausal period examined in our study. Although this study was observational, it provides important supportive evidence for the biological plausibility of our interventional findings, reinforcing the role of vitamin D in neuromuscular function and central nervous system regulation. Taken together, these studies provide a coherent framework supporting the results of the present research. While not all trials report uniform benefits, the majority of studies conducted in vitamin D deficient and symptomatic populations demonstrate significant improvements in mood and fatigue following supplementation. Our study adds to this evidence by specifically focusing on perimenopausal women, a group particularly susceptible to fatigue and mood disturbances due to hormonal fluctuations and nutritional deficiencies. The consistency of our findings with several prior PubMed-indexed studies strengthens the argument for considering vitamin D supplementation as a simple, safe and adjunctive therapeutic strategy for managing fatigue and mood symptoms during the perimenopausal transition.

## Conclusion:

Vitamin D supplementation significantly improved fatigue and mood symptoms in perimenopausal women with vitamin D deficiency, along with a marked rise in serum vitamin D levels. These benefits were sustained over six months, suggesting an important role for vitamin D in neuromuscular and psychological well-being during the perimenopausal transition. Vitamin D supplementation may therefore be a safe, affordable, and accessible adjunctive therapeutic option in this population, although larger studies are needed to confirm these findings and define optimal dosing.

## Limitations:

The present study has several limitations that should be acknowledged. First, the relatively small sample size of 100 participants and the single-center design may limit the generalizability of the findings to broader perimenopausal populations with different socioeconomic, ethnic and lifestyle backgrounds. Second, fatigue and mood were assessed using self-reported questionnaires, which are inherently subjective and may be influenced by recall bias or social desirability bias. Third, although major confounders such as age and BMI were adjusted for, other potential influencing factors including dietary intake, physical activity, sleep quality, stress levels and seasonal variation in sun exposure were not rigorously controlled. Fourth, the study duration of six months may not be sufficient to determine the long-term sustainability of the observed benefits of vitamin D supplementation. Finally, serum vitamin D levels were measured only at baseline and at study completion, which limited the ability to assess temporal fluctuations and dose response relationships. These limitations suggest that larger, multicenter trials with longer follow-up and more comprehensive control of confounding variables are needed to confirm and extend the present findings.
